# Supplementary figures and images for: Novel Insight into the Potential Role of Acylglycerophosphate Acyltransferases Family Members on Triacylglycerols Synthesis in Buffalo
Source: Int J Mol Sci. 2022 Jun 12;23(12):6561. doi: 10.3390/ijms23126561 (PMC9224252; doi:10.3390/ijms23126561)

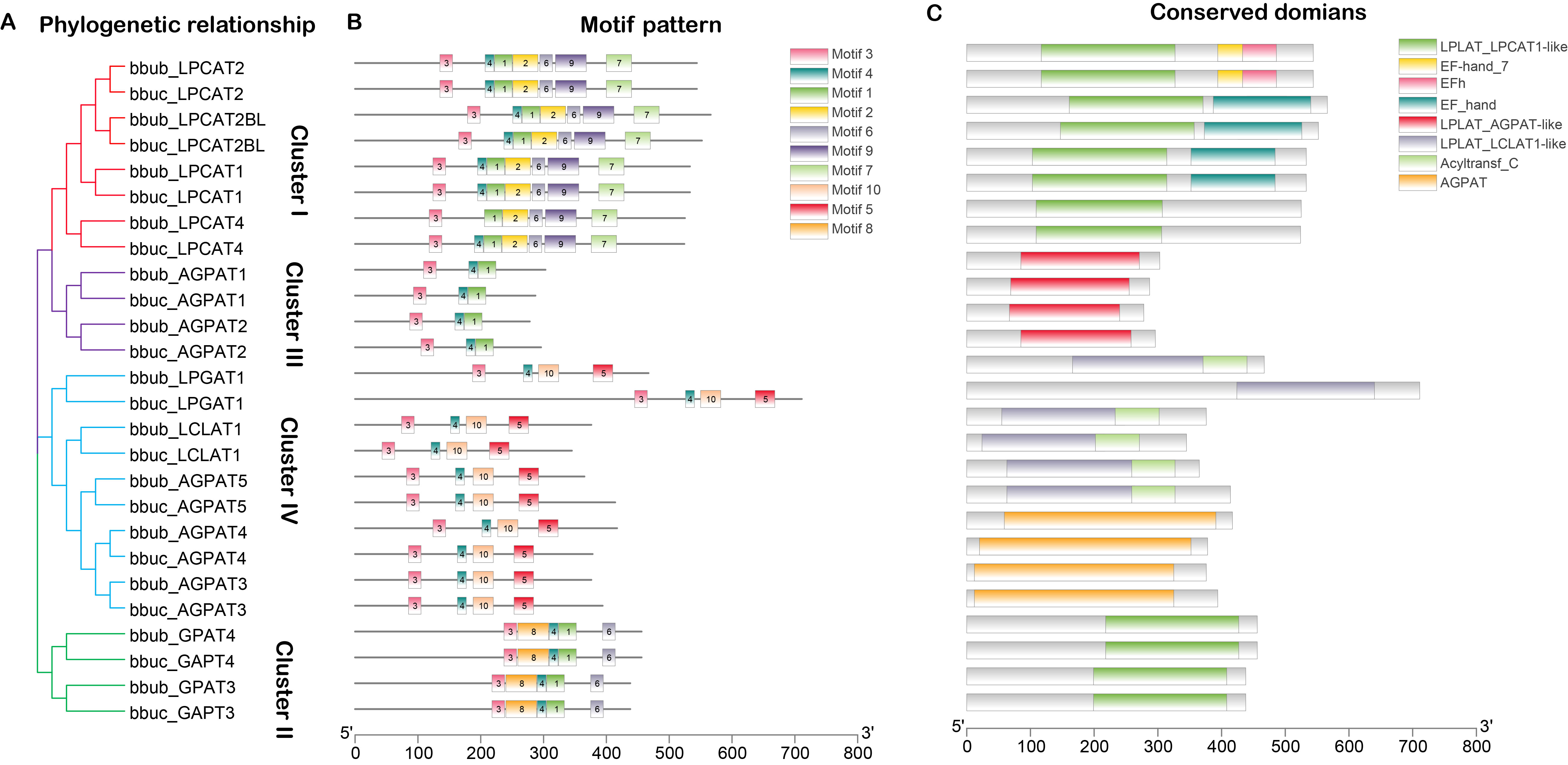

Supplement: Supplementary file 1 [file ijms-23-06561-s001.zip › Figure S1.jpg]

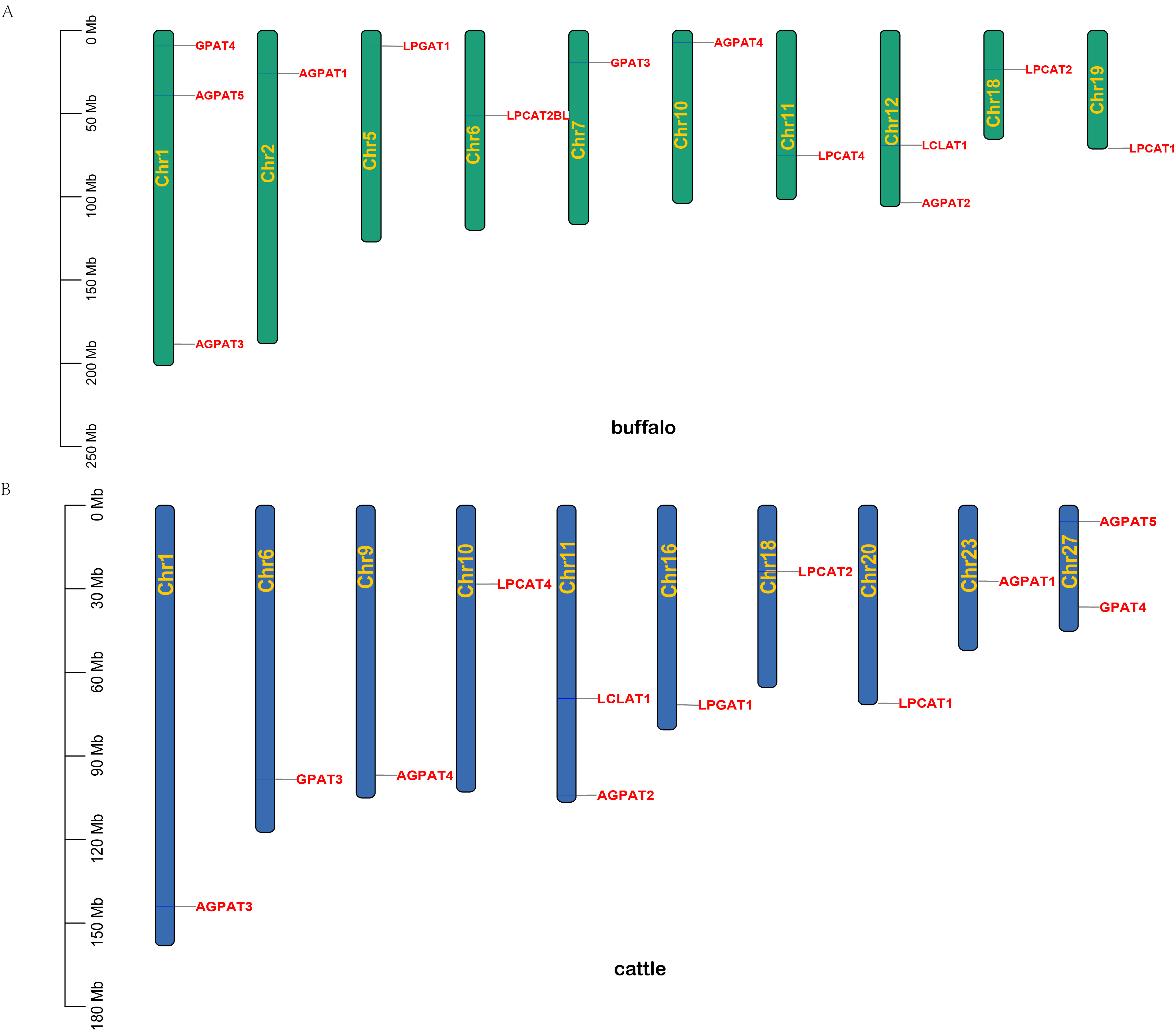

Supplement: Supplementary file 1 [file ijms-23-06561-s001.zip › Figure S2.jpg]
